# Radon Solubility and Diffusion in the Skin Surface Layer

**DOI:** 10.3390/ijerph19137761

**Published:** 2022-06-24

**Authors:** Akihiro Sakoda, Tsuyoshi Ishida, Norie Kanzaki, Hiroshi Tanaka, Takahiro Kataoka, Fumihiro Mitsunobu, Kiyonori Yamaoka

**Affiliations:** 1 Ningyo-toge Environmental Engineering Center, Japan Atomic Energy Agency, 1550 Kamisaibara, Kagamino-cho, Tomata-gun, Okayama 708-0698, Japan; kanzaki.norie@jaea.go.jp (N.K.); tanaka.hiroshi00@jaea.go.jp (H.T.); 2 Graduate School of Health Sciences, Okayama University, 5-1 Shikata-cho 2-chome, Kita-ku, Okayama 700-8558, Japan; ishida.tsuyoshi@jaea.go.jp (T.I.); kataokat@md.okayama-u.ac.jp (T.K.); yamaoka@md.okayama-u.ac.jp (K.Y.); 3 Graduate School of Medicine Dentistry and Pharmaceutical Sciences, Okayama University, 5-1 Shikata-cho 2-chome, Kita-ku, Okayama 700-8558, Japan; fumin@cc.okayama-u.ac.jp

**Keywords:** radon, partition coefficient, diffusion, biokinetic model, skin, sebaceous lipids, stratum corneum, viable skin

## Abstract

In specific situations such as bathing in a radon spa, where the radon activity concentration in thermal water is far higher than that in air, it has been revealed that radon uptake via skin can occur and should be considered for more precise dose evaluation. The primary aim of the present study was to numerically demonstrate the distribution as well as the degree of diffusion of radon in the skin, with a focus on its surface layer (i.e., stratum corneum). We developed a biokinetic model that included diffusion theory at the stratum corneum, and measured radon solubility in that tissue layer as a crucial parameter. The implementation of the model suggested that the diffusion coefficient in the stratum corneum was as low as general radon-proof sheets. After a 20-min immersion in water, the simulated depth profile of radon in the skin showed that the radon activity concentration at the top surface skin layer was approximately 10^3^ times higher than that at the viable skin layer. The information on the position of radon as a radiation source would contribute to special dose evaluation where specific target cell layers are assumed for the skin.

## 1. Introduction

We are constantly exposed to ionizing radiation arising from naturally occurring sources, e.g., radiation from radon (^222^Rn)—a radioactive inert gas—emanates from soil and building materials. For decades, the international agreement has been that the annual global average exposure to natural radiation sources for members of the public is 2.4 mSv, with radon exposure accounting for half of that [1,2]. To be more precise, this radon exposure corresponds with the inhalation of not radon itself but rather its short-lived progeny. On the other hand, the importance of other exposure pathways has been argued for special circumstances such as radon spas: i.e., the inhalation of radon itself, the transfer of radon via the skin, and the deposition of radon progeny on the skin.

Making model structures and their related parameterization for such pathways has recently been enhanced to better mimic empirical data from human research on their activity concentrations in organs and tissues as well as on the skin surface during or after the exposure. Using convenient assumptions as well as parameters such as a skin permeability coefficient defined in [3,4], the models can work for purposes dedicated to generic dose assessments from the viewpoint of radiation protection or therapeutic application [5,6]. There is still a lot of work to be done in terms of understanding and describing the behavior of radon and its progeny at the skin. The medium–skin contact of radon or its progeny, followed by its transfer to the viable epidermis and dermis (called hereinafter as “viable skin”) as the bloodstream entrance through the stratum corneum, which serves as a skin barrier, should be better understood.

Given the fact that there are no implications on its distribution in skin tissues, we addressed the development of a biokinetic model of radon incorporating the diffusion theory. We also measured the partition coefficient between the stratum corneum and water, which is a model parameter that represents radon solubility in the skin surface. It is worth noting that radon solubility in sebaceous lipids is a beginning process of radon uptake via the skin because it is rather soluble in fatty substances [7,8]. Finally, the diffusion coefficient of radon in the stratum corneum was determined by fitting the model calculation to earlier human data. This modeling work would be the first step in determining the depth profile of radon in the skin, paving the way for more advanced dosimetry that takes into account specific target cells or layers.

## 2. Materials and Methods

### 2.1. Measurement of Radon Partition Coefficients

Human sebum mainly consists of triglycerides (TG), free fatty acids (FFA), wax esters (WE), and squalene (SQ) [9,10]. Four lipid component samples (FUJIFILM Wako Pure Chemical Corporation, Japan) were used for this study: i.e., triolein (C_57_H_104_O_6_ = 885.43; 0.91 g mL^−1^) as a TG, oleic acid (CH_3_(CH_2_)_7_CH:CH(CH_2_)_7_COOH = 282.46; 0.89 g mL^−1^) as an FFA, methyl myristate (CH_3_(CH_2_)_12_COOCH_3_ = 242.40; 0.87 g mL^−1^) as a WE, and squalene (C_30_H_50_ = 410.72; 0.86 g mL^−1^ at 20 °C). To produce a simplified model of sebum, triolein (43% by volume), oleic acid (18%), methyl myristate (26%), and squalene (13%) were combined together.

Radon-rich water (about 2000–4000 Bq l^−1^) was first produced by circulating radon-rich air (about 1 MBq m^−3^) through distilled water [11]. Using a water bath, the temperatures of the water and lipid samples were set to 20, 30, or 40 °C. Then, using a syringe and needle, 30 mL of radon-rich water was gently injected into the bottom of a 50-mL centrifuge tube, which contained 10 mL of lipid sample and 15 mL of radon-free air. The centrifuge tube was tightly closed and placed in a shaking water bath for 5 min at temperatures of 20, 30, or 40 °C, with a frequency of 140 per second. After that, 5 mL of the lipid sample taken by penetrating a needle to the wall of the centrifuge tube was put in an airtight 5-mL vial. In addition, about 25 mL of the water sample taken from the centrifuge tube was placed in a 110-mL vial that had already contained 20 mL of the toluene-based scintillator.

Radon activity concentration in the lipid sample, *C*_lipid_ (Bq l^−1^), was measured by gamma-ray counting from ^214^Pb and ^214^Bi for 60 min with a high-purity germanium detector (GWL-120-15, ORTEC). To achieve a radioactive equilibrium between radon and its progeny, it takes at least 4 h after sealing the vial. On the other hand, radon activity concentration in the water sample, *C*_water_ (Bq l^−1^), was measured by alpha- and beta-ray counting from ^222^Rn, ^218^Po, ^214^Pb, ^214^Bi, and ^214^Po for 10 min with a liquid scintillation counter (LSC-LB5, ALOKA, Japan). More than 4 h before beginning the measurement, the sample vial was hand-shaken for 30 s to ensure the equilibrium of radon activity concentrations among the liquid scintillator, water, and air. Despite the different measurement methods taken for the lipid and water samples, both methods are well established and did not require any correction to determine partition coefficients as defined below.

The partition coefficient of radon, *P*_sample/air_ (−), is defined as

(1) $$P_{\mathrm{sample}/\mathrm{air}}=\frac{C_{\mathrm{sample}}}{C_{\mathrm{air}}},$$

where *C*_sample_ and *C*_air_ are the radon activity concentrations in a specific substance (e.g., lipid and water) and air, respectively. Here, *C*_air_ is unknown in this investigation, as radon in the air phase of the conditioned centrifuge tube is not measured. Instead, *C*_air_ was calculated using our measured *C*_water_ and the empirical formula [12]:

(2) $$C_{\mathrm{air}}=\frac{C_{\mathrm{water}}}{P_{\mathrm{water}/\mathrm{air}}}=\frac{C_{\mathrm{water}}}{0.105+0.405e^{-0.0502T}},$$

where *T* (°C) is temperature.

### 2.2. Biokinetic Model for Inhaled and Skin-Absorbed Radon

**Figure 1:**
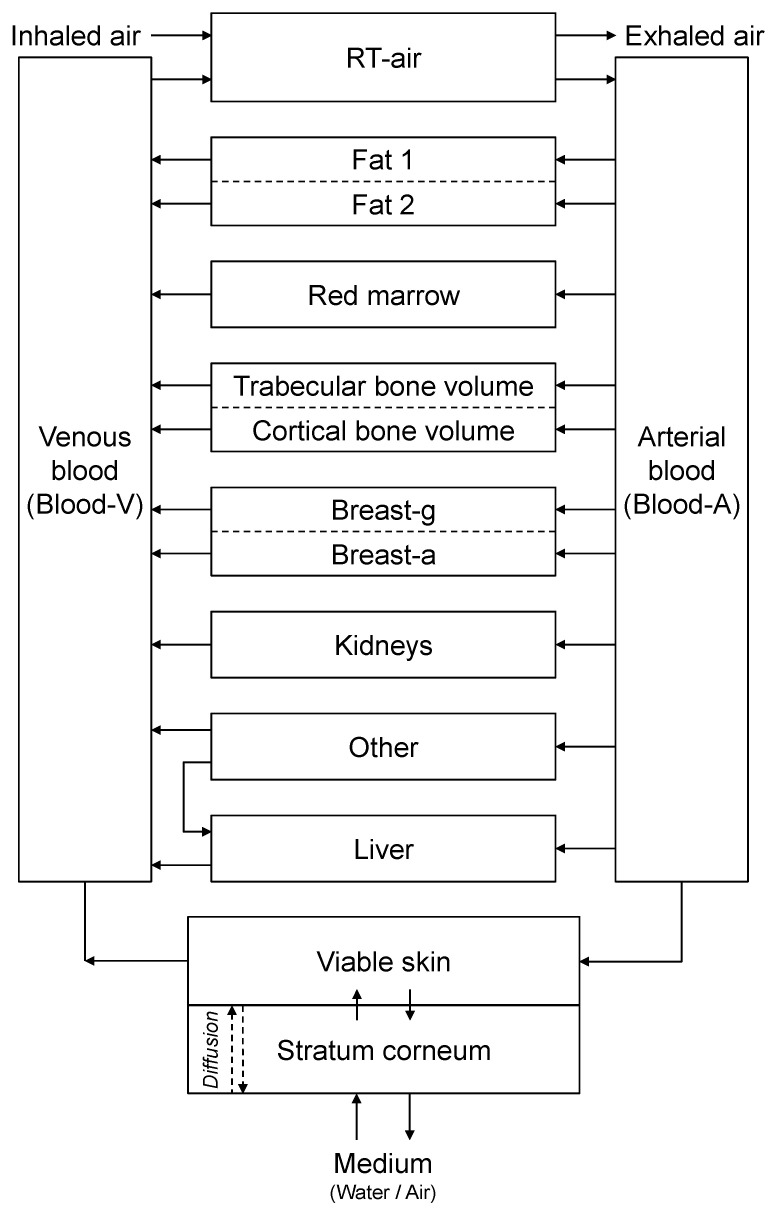
Schematic diagram of the biokinetic model for inhaled or skin absorbed radon. RT-air, respiratory air; Breast-g, glandular tissue of breast; Breast-a, adipose tissue of breast. The fundamental mathematical description is explained in [4]. The movement of radon in the compartment “Stratum corneum” is expressed by the diffusion equation (see the text).

Figure 1 shows the biokinetic model used in this work. This model was built by adding skin compartments to a generic model for noble gases published by Leggett et al. [13]. The current model can deal with radon uptake through the skin in the same way as our earlier model [4], with the exception that the former is a distributed-parameter model and the latter is a lumped-parameter model [14] in terms of skin compartments.

In the present study, therefore, the transport of radon within the outermost skin layer (i.e., stratum corneum)—the process “Diffusion” in Figure 1—in contact with water including radon is expressed by the one-dimensional diffusion equation. The mathematical treatment describing the biokinetics of radon within the body—an activity balance at each compartment—was the same as that which Leggett et al. [13] and the authors [4] used previously:

(3) $$\frac{\partial C_{\mathrm{SC}}}{\partial t}=D_{\mathrm{SC}}\frac{\partial^{2}C_{\mathrm{SC}}}{\partial z^{2}},$$

where *C*_SC_ (Bq m^−3^) is the radon activity concentration, *D*_SC_ (m^2^ s^−1^) is the diffusion coefficient of radon, and the subscript “SC” stands for the compartment “Stratum corneum”. To simplify the numerical calculation and achieve an approximate answer, the compartment “Stratum corneum” was discretized into *N* + 2 equidistant nodes; *N* = 100 was used in this study. As a result, the following formulas can be used to calculate the concentration profile:

(4) $$\frac{dC_{\mathrm{SC},i}}{dt}=D_{\mathrm{SC}}\frac{\left(C_{\mathrm{SC},i+1}-C_{\mathrm{SC},i}\right)-\left(C_{\mathrm{SC},i}-C_{\mathrm{SC},i-1}\right)}{{\left(L_{\mathrm{SC}}/\left(N+1\right)\right)}^{2}}-\lambda C_{\mathrm{SC},i}\;\;\;\;\;\;\;\;\;\;i=1,2\cdots N,$$

where *L*_SC_ (m) is the diffusion pathlength of radon in the stratum corneum and *λ* (s^−1^) is the decay constant of radon. The concentrations at the two extreme nodes (*i* = 0 and *N* + 1) are given by the boundary conditions:

(5) $$C_{\mathrm{SC},0}=C_{\mathrm{medium}}P_{\mathrm{SC}/\mathrm{medium}}$$

and

(6) $$C_{\mathrm{SC},N+1}=\frac{C_{\mathrm{VS}}}{P_{\mathrm{VS}/\mathrm{SC}}},$$

where *C*_medium_ and *C*_VS_ (Bq m^−3^) are the radon activity concentrations, *P*_SC/medium,_ and *P*_VS/SC_ (–) are the partition coefficients of radon, and the subscript “VS” stands for the compartment “Viable skin.” Also, the subscript “medium” can be replaced with “water” or “air.” The change in *C*_VS_ for body parts other than the head or the head part is given by

(7) $$V_{\mathrm{VS}}\frac{dC_{\mathrm{VS}}}{dt}=F_{\mathrm{skin}}\left(C_{A}-\frac{C_{\mathrm{VS}}}{P_{\mathrm{skin}/\mathrm{blood}}}\right)-\lambda V_{\mathrm{VS}}C_{\mathrm{VS}}+J_{\mathrm{SC},N+1}A_{\mathrm{skin}},$$

where *V*_VS_ (m^3^) is the volume of the viable skin in body parts other than the head or the head part, *F*_skin_ (m^3^ s^−1^) is the blood flow rate for the skin in body parts other than the head or the head part, respectively, *C*_A_ (Bq m^−3^) is the radon activity concentration in non-pulmonary arterial blood, *P*_skin/blood_ (–) is the skin-to-blood partition coefficient of radon, *J*_SC__,*N*+1_ is the radon flux at the interface of the stratum corneum and viable skin, and *A*_skin_ (m^2^) is the skin surface area of other than the head or the head. *J_SC,N_*_+1_ can be calculated and approximated by

(8) $$J_{\mathrm{SC},N+1}=-D_{\mathrm{SC}}\frac{dC_{\mathrm{SC},N+1}}{dz}=-D_{\mathrm{SC}}\left(\frac{C_{\mathrm{SC},N+1}-C_{\mathrm{SC},N}}{L_{\mathrm{SC}}/\left(N+1\right)}\right).$$

Equations (4)–(8) were formulated separately for each skin part (i.e., other than the head or the head), whereas the same *D*_SC_ was applied to both parts.

Table 1 gives the parameter values used in Equations (4)–(8). *P*_SC/water_ was determined based on the experimental result from Section 2.1, assuming that the sebaceous lipid covers the body surface. Other parameter values necessary for the biokinetic model were taken from the refs. [4,13,15].

Our model calculation data were fitted to published human data [16,17,18] on radon activity concentrations in breathed air to determine the values and range of *D*_SC_. Except for the head, each subject’s body was assumed to be immersed in thermal water during the experiment. The subjects’ exhaled air samples were collected during and/or after bathing, and radon activity concentrations were assessed. The study of Nagy [16] used radon-free air for breathing, meaning that the skin was the only compartment for the introduction of radon. The experimental circumstances in the research of Tempfer et al. [17] and Furuno [18] were similar, although the radon activity concentrations in water and air were different by an order of magnitude.

## 3. Results

**Figure 2:**
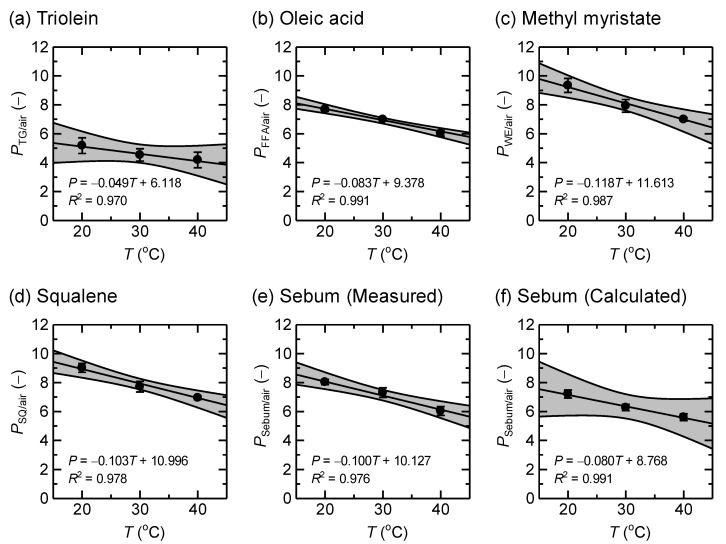
Partition coefficients of radon for sebaceous lipid samples as a function of temperature. Solid lines stand for the linear regression curves with 95% confidence intervals. The linear regression analysis was implemented for all individual data (*n* = 3 at each temperature).

Table 2 presents the partition coefficients (*P*_sample/air_) of radon for sebaceous lipid samples. The measured *P*_sample/air_ can be sorted as WE ≈ SQ > FFA > TG. The range for the four samples was 4.28–7.26 at 37 °C, and the measured and calculated values of *P*_sample/air_ for the sebum sample were in general agreement considering the 95% confidence interval. In the biokinetic model calculation, the measured *P*_sebum/air_ of 6.43 at 37 °C was used as *P*_SC/air_. Figure 2 visualizes the measured data of Table 2 to illustrate the liner decrease of *P*_sample/air_ with increasing temperature. Although another (non-linear) function may be available, linear regression was practically the best for this result. In comparison to other efforts [12,19,20], the non-linear regression did not produce reasonable and coherent fitted parameters despite a good fit. This is most likely because we only observed three points (20, 30, and 40 °C) in our experiment, and the temperature range was too narrow to apply a more sophisticated function.

**Figure 3:**
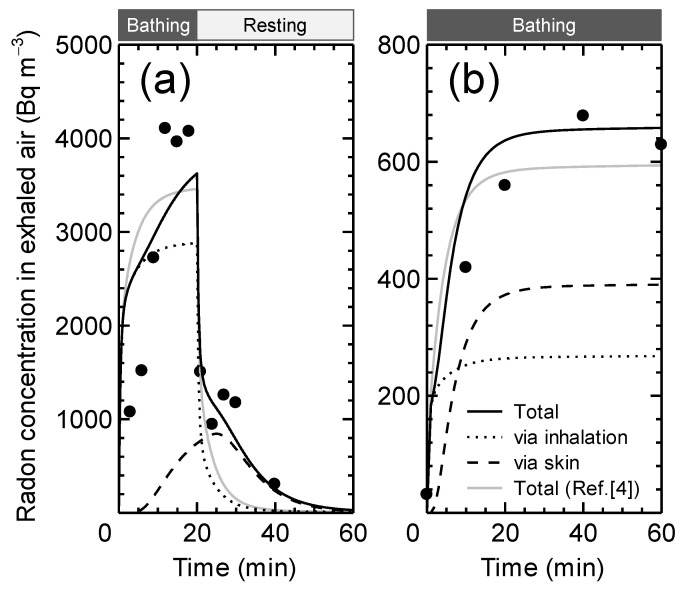
Comparison of radon activity concentrations in exhaled air during and after immersion in thermal water between model estimates and human experiments. Experimental data: (**a**) Tempfer et al. [17] and (**b**) Furuno [18]. The exposure conditions are summarized in Table 3. The solid curves were obtained by the use of the best estimated *D*_SC_ values. The curves (“Total”) were then divided into inhalation and dermal uptake, taking into consideration the contribution of each exposure route. The best-fitted data given by the previous model are depicted as well [4].

Figure 3 depicts the best fit of the computed data to the experimental data, which indicates changes in radon activity concentrations in exhaled air samples during or after exposure. Table 3 shows the fitted *D*_SC_ values based on Nagy’s human investigation [16], as well as those of Tempfer et al. [17] and Furuno [18] (Figure 3). The range of *D*_SC_ was found to be mostly within the orders of 10^−14^ and 10^−13^ m^2^ s^−1^. It should also be noted that *D*_SC_ fitted to Nagy’s data had a log-normal distribution with a geometric mean of 6.9 × 10^−14^ m^2^ s^−1^ and a geometric standard deviation of 3.3, as is the similar distribution to skin permeability coefficients defined in a previous modeling study [4]. The relationship between *D_SC_* (m^2^ s^−1^) and the skin permeability coefficient *K* (m s^−1^) can be approximated as *D*_SC_ = 3 × 10^−7^ *K*.

**Figure 4:**
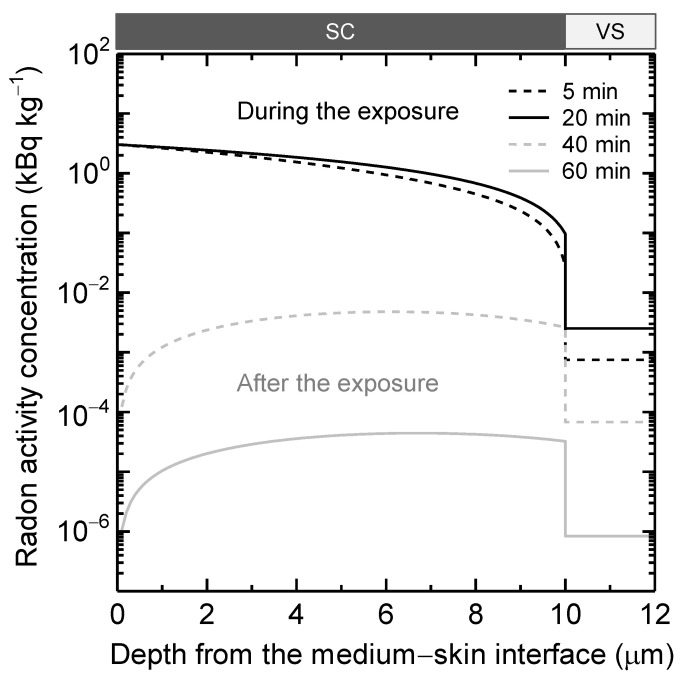
Radon activity concentrations in the skin at different depths during and after immersion in thermal water. The following were the radon exposure parameters: 0–20 min with the exposure only from thermal water (100 Bq l^−1^; 37 °C); 20–60 min without exposure from thermal water and air; male subject; *D*_SC_ = 6.4 × 10^−14^ m^2^ s^−1^ (median of *D*_SC_ for males, assessed from the human study of Nagy [16]). The stratum corneum (SC) was assumed to be 10-μm thick, and the deeper part was the viable skin (VS), where the radon activity concentration was assumed to be uniform, with a typical thickness of 1000–2000 μm [15] that is not used as an input parameter for the modeling.

Figure 4 depicts the radon activity concentrations in the skin at different depths during or after the exposure. The differences in curve morphologies between the stratum corneum (SC) and viable skin (VS) are related to the mathematical treatment used in the model; in particular, the activity balance in the entire VS was stated using a single compartment, rather than reflecting diffusion-based movement as in the SC. Even within 5 min, radon was clearly collected in the skin. Also, the radon activity concentrations at *z* = 10 μm (i.e., the bottom of SC) and *z* > 10 μm are lower by factors of >10 and >10^3^, respectively, than that at *z* = 0 μm. After the exposure, in contrast, the opposite shape of the curves for SC is seen, resulting from the significant removal of radon to air from the skin. The radon activity concentration at *z* > 10 μm is lower by factors of >10 than that at *z* = 10 μm; here, it should be noted that the model assumed the concentration *C*_SC,0_ = 0 Bq m^−3^ (=*C*_air_) at *z* = 0 μm, as defined in Equation (5).

## 4. Discussion

The biokinetic model for radon was developed to examine its heterogeneous distribution in the stratum corneum. To run this model, the partition coefficients of radon for the sebum and its main components had to be determined experimentally first and then *P*_sebum/air_ was assigned to *P*_SC/air_ according to other modeling works (e.g., [14]). Nussbaum and Hursh measured *P*_sample/air_ for fatty acids and triglycerides as being at 37 °C, 0.96 for formic acid, 2.88 for triacetin, 3.53 for acetic acid, and 5.01–7.23 for more than 20 substances including oleic acid [8]. Our *P*_sample/air_ values for the sebaceous lipid samples (Table 2) were mostly within this main range, except for *P*_TG/air_, which was outside of and a bit below it. Our value (6.32) for oleic acid was determined to be similar to the reported value (6.72) with a discrepancy rate of 6% (despite being out of the 95% confidence interval, 6.06–6.59, as shown in Table 2), indicating that the current experiment was properly conducted. Furthermore, Ishimori et al. also measured and approximated *P*_sample/air_ for mouse tissue and organs, to which we corrected their original report of *P*_sample/blood_ to: 0.091 for brain, lung, kidney, pancreas, guts; 0.19 for muscle; 0.31 for liver; 0.414 for blood; and 3.47 for adipose tissue [11]. The sebaceous lipids were found to have a significantly higher *P*_sample/air_ than the major organs and tissues.

Time-course changes in radon exhaled by the breath during the exposure (Figure 3), including the trend of the sharper rise at the initial phase (0–5 min) that is attributable to the inhalation rather than the skin absorption, were similar between the current and earlier models. It was also shown that the current model takes longer than the previous model to reach saturation of radon activity concentrations, which is noticeable in the case of low *D*_SC_ (see Figure 3a). This is the reflection of the incorporation of the diffusion process through the stratum corneum. Likewise, the present model takes more time to remove radon from the body via both the breath and skin after the exposure, resulting in the better fit of the calculated data to the human data during the rest time (Figure 3a). Radon retained in the stratum corneum influences the gradual decline in radon activity concentrations in exhaled air. If the *D*_SC_ is substantially lower or the *L*_SC_ is thicker, a much slower drop in radon activity concentration in exhaled air or the formation of a peak can be reasonably expected, as shown in the literature [14].

The values of *D*_SC_ estimated here ranged from 9.7 × 10^−15^ to 5.0 × 10^−^^13^ m^2^ s^−^^1^, covering 90% of its log-normal distribution. Such *D*_SC_ values are far lower than the molecular diffusion coefficients of radon in the air (10^−5^ m^2^ s^−1^) and water (10^−9^ m^2^ s^−1^) [21], and are lower by a few orders of magnitude than or as low as radon diffusion coefficients for radon-proof sheets: e.g., in the order of 10^−14^ m^2^ s^−^^1^ for ethylene vinyl acetate (EVA), in the order of 10^−13^ m^2^ s^−^^1^ for bitumen with aluminum film, in the order of 10^−12^ m^2^ s^−^^1^ for aluminum foil, high-density polyethylene (HDPE), mylar film, polypropylene (PP); 10^−11^ m^2^ s^−^^1^ for low-density polyethylene (LDPE) and polyvinyl chloride (PVC) [22,23].

The radon diffusion coefficient was quantified in our investigation utilizing earlier human studies rather than well-conditioned laboratory tests (as in the previous studies using commercially available materials). As a result, we need to include more elements of uncertainty, one of which is the physiological or anatomical parameters used in the biokinetic model computation due to individual differences in human beings. Here, it should be discussed how the different assumption of the stratum corneum thickness (*L*_SC_ = 10 μm in this study) influences the result of *D*_SC_. If *L*_SC_ was assumed to be 10 or 20 μm (in the case of all male subjects), the analysis of the Nagy’s human data [16] yielded the following *D*_SC_: for *L*_SC_ = 10 μm, GM 6.4 × 10^−^^14^ m^2^ s^−^^1^ (GSD = 3.3), 5th percentile 9.1 × 10^−^^15^ m^2^ s^−^^1^, and 95th percentile 4.6 × 10^−^^13^ m^2^ s^−^^1^; for *L*_SC_ = 20 μm, GM 1.3 × 10^−^^13^ m^2^ s^−^^1^ (GSD 3.3), 5th percentile 1.9 × 10^−^^14^ m^2^ s^−^^1^, and 95th percentile 9.1 × 10^−^^13^ m^2^ s^−^^1^. This means that even if one uses a possible different assumption of *L*_SC_—maybe selected from 7–15 μm which is the usual stratum corneum thickness for normally clothed regions of the body [15], the resulting *D*_SC_ is not orders of magnitude more or less than that for the case of *L*_SC_ = 10 μm. At the same time, it should also be added that radon is not exhaled via breathing when *L*_SC_ ≥ 600 μm (equivalent to *L*_SC_ at the palms and soles [15]) and *D*_SC_ ≤ 10^−^^12^ m^2^ s^−^^1^ (covering more than 95% of intersubject variability as shown in Table 3) are assumed. This is because radon can hardly penetrate such thick stratum corneum.

Furthermore, the model may have assumed two conditions: that the stratum corneum layer underneath the top layer is uniform, and that the lipid layer is just at the skin’s surface. The former appears to be realistic as there are three possible paths by which permeant substances may pass through the stratum corneum: i.e., the lipid matrix, keratinized cells, and the shunt pathway via hair follicles or sweat glands [14]. For dermal absorption of organic chemicals, the lipid matrix pathway is considered to be the predominant route. When it comes to radon, the argument on such pathways, which is beyond the scope of the present work, is important for better understanding of the underlying mechanisms and requires further research.

On the other hand, the latter appears to be an extreme situation whose impact should be debated. That is, it will be examined how *D*_SC_ is changed if not *P*_SC/water_ = *P*_sebum/water_ = 33.3 (Table 1) but *P*_SC/water_ = *P*_skin/water_ = *P*_skin/blood_ *P*_blood/air_ *P*_air/water_ = 0.4 × 0.43 × 5.2 = 0.89 (i.e., another extreme assumption) is assigned to Equation (5). If *P*_SC/water_ was considered to be 33.3 or 0.89 (in the case of all male subjects), the examination of Nagy’s human data [16] revealed the following *D*_SC_: for *P*_SC/water_ = 33.3, the same as the case for *L*_SC_ = 10 μm shown in the above paragraph; for *P*_SC/water_ = 0.89, GM 2.4 × 10^−12^ m^2^ s^−1^ (GSD = 3.4), 5th percentile 3.2 × 10^−13^ m^2^ s^−1^, and 95th percentile 1.8 × 10^−11^ m^2^ s^−1^. The lower *P*_SC/water_ indicates the lower radon activity concentration at the skin surface, resulting in at most two orders of magnitude higher *D*_SC_. Nonetheless, the *D*_SC_ range obtained with *P*_SC/water_ = 0.89 may still be classed as being as low as the radon-proof sheets (i.e., 10^–14^ to 10^–11^ m^2^ s^−1^) mentioned before. This argument is also helpful to interpret how the *D*_SC_ estimate is affected by different compositions of the sebum (i.e., different *P*_SC/water_).

The present modeling potentially assumed no dependence of *D*_SC_ on water temperature, while considering that *P*_SC/water_ is slightly inversely proportional to temperature from 20 to 40 °C. This means that the simulated radon activity concentrations in exhaled air decreases with increasing water temperature. In contrast, Hofmann et al. [3] experimentally revealed a significant increase in the exhaled radon activity concentrations in the same temperature range. The discrepancy between the computed and measured results cannot be solved in the present paper but might suggest that the effect of water temperature on *D*_SC_ should be introduced into the model. In the future, therefore, the model analysis of radon exhalation curves in many human subjects reported by Hofmann et al. [3] will be performed to associate *D*_SC_ with temperature and to discuss the reliability of Nagy’s data [16] that consisted of a single measurement for each subject (Table 3) and the reasonableness of the intersubject variability of *D*_SC_ estimated from her data.

Finally, the distribution of radon in the skin is discussed; the outline of the depth profile was already illustrated in the Results section. A lot more radon was rapidly accumulated in the stratum corneum than in the viable skin (Figure 4). However, it should also be noted that the radon activity concentration in the viable skin was much higher than in other organs and tissues depicted in Figure 1. For example, at the end of the exposure (*t* = 20 min), the radon activity concentrations were calculated as follows: 9.7 × 10^−2^ to 3 kBq kg^−1^ for the stratum corneum, 2.5 × 10^−3^ kBq kg^−1^ for the viable skin, 1.1 × 10^−4^ kBq kg^−1^ for Blood-A, 4.0 × 10^−4^ kBq kg^−1^ for Blood-V, 8.7 × 10^−6^–1.2 × 10^−4^ kBq kg^−1^ for the other organ/tissue compartments. In this case, the concentration in the viable skin was >10 times higher than that in the other organs and tissues (except for the stratum corneum).

Although the mobility of radon decay products should be explored further in the future, the current study provides important information on the position of radon as a radiation source for special dose evaluation when specific target cell layers are assumed for the skin. Basal cells positioned at the bottom layer of the epidermis and Langerhans cells existing uniformly in the epidermis have been considered as target cells from the viewpoint of radiation risk and radon spa therapy, respectively [5,6]. It would be fascinating to learn more about how radon is distributed within the skin (in particular, the viable skin), which could help with dose evaluation. This necessitates a simulation of the radon activity balances between the epidermis/dermis layer and the stratum corneum or blood, as well as radon diffusion movement in the epidermis and dermis. At the same time, more in vitro and in vivo studies may be desirable to validate such models.

## 5. Conclusions

The current research quantified the entry of radon into the skin as well as its distribution during and after exposure. To develop the biokinetic model of radon that incorporates diffusion theory at the stratum corneum, we first experimentally yielded the partition coefficients of radon for the sebum and its components, which were found to be mostly within the range of previously reported partition coefficients for fatty acids and triglycerides. The model was then implemented with the obtained *P*_sebum/air_, suggesting that *D*_SC_ was as low as radon-proof sheets (i.e., the order of 10^−14^ to 10^−11^ m^2^ s^−1^) tested in previous studies, even if the uncertainty of *L*_SC_ and *P*_SC/air_ was considered. In addition, the calculation of the depth profile of radon in the skin surface represented that *C*_SC,0_ was about 10^3^ times greater than *C*_VS_ after exposure for 20 min in water.

The knowledge on radon’s position as a radiation source would help with particular dose evaluations in which specific target cell layers for the skin are assumed: basal cells and Langerhans cells from the perspectives of radiation risk and radon spa therapy, respectively. In addition to the stratum corneum, understanding the distribution of radon in the viable epidermis and dermis, which is an issue to be examined in the future, may improve this kind of dose evaluation.

## Figures and Tables

**Table 1:** Parameters necessary for modeling the diffusion of radon in the stratum corneum.

Parameter		Value	Reference
*A*_skin_ (m^2^)	Skin surface area for other than the head	17,575 (male) 15,355 (female)	Calculated from [15].
	Skin surface area for the head	1425 (male) 1245 (female)	
*F*_skin_ (m^3^ s^−1^)	Blood flow rate in the skin for other than the head	5.01 × 10^−6^ (male) 4.55 × 10^−6^ (female)	Calculated from [15].
	Blood flow rate in the skin for the head	4.06 × 10^−7^ (male) 3.69 × 10^−7^ (female)	
*L*_SC_ (m)	Thickness of the stratum corneum	10 × 10^−6^ (for normally clothed regions of the body)	Approximated from [15].
*P*_SC/water_ (dimensionless)	SC-to-water partition coefficient	33.3 (at 37 °C)	Calculated from *P*_SC/air_ = 6.43 (see the text and Table 2) and *P*_air/water_ = 5.17.
*P*_skin/blood_ (dimensionless)	Skin-to-blood partition coefficient	0.4	Taken from [13].
*P*_VS/SC_ (dimensionless)	VS-to-SC partition coefficient	0.0267	$\mathrm{Calculated}\;\mathrm{by}\;\frac{P_{\mathrm{skin}/\mathrm{blood}}P_{\mathrm{blood}/\mathrm{air}}P_{\mathrm{air}/\mathrm{water}}}{P_{\mathrm{SC}/\mathrm{water}}}.$
*V*_VS_ (m^3^)	Volume of the viable skin for other than the head	2.61 × 10^−3^ (male) 1.82 × 10^−3^ (female)	Calculated from [15].
	Volume of the viable skin for the head	2.85 × 10^−4^ (male) 1.98 × 10^−4^ (female)	

Note: The subscripts “SC” and “VS” stand for the stratum corneum and viable skin, respectively.

**Table 2:** Measured partition coefficients of radon for sebaceous lipid samples.

Sample		*P*_sample/air_ (−)			
		20 °C	30 °C	37 °C ^a^	40 °C
Triolein (TG)		5.18 ± 0.55 (4.08, 6.16)	4.54 ± 0.45 (3.99, 5.26)	4.28 (3.42, 5.14)	4.19 ± 0.54 (3.09, 5.17)
Oleic acid (FFA)		7.68 ± 0.13 (7.41, 8.04)	6.99 ± 0.11 (6.70, 7.10)	6.32 (6.06, 6.59)	6.03 ± 0.20 (5.76, 6.39)
Methyl myristate (WE)		9.34 ± 0.48 (8.49, 10.4)	7.93 ± 0.43 (7.60, 8.58)	7.26 (6.62, 7.91)	6.99 ± 0.11 (6.14, 7.69)
Squalene (SQ)		9.02 ± 0.31 (8.33, 9.52)	7.72 ± 0.36 (7.52, 8.27)	7.17 (6.67, 7.66)	6.95 ± 0.13 (6.27, 7.45)
Sebum ^b^	Measured	8.04 ± 0.18 (7.54, 8.71)	7.31 ± 0.33 (6.76, 7.50)	6.43 (5.94, 6.92)	6.04 ± 0.30 (5.54, 6.71)
	Calculated ^c^	7.21 ± 0.27 (5.71, 8.59)	6.27 ± 0.22 (5.51, 7.20)	5.80 (4.73, 6.87)	5.61 ± 0.24 (4.26, 6.86)

Note: The values in the parentheses represent 95% confidence intervals given by the linear regression in Figure 2. ^a^ Fitted values from Figure 1. ^b^ Composition: TG 43%, FFA 18%, WE 26%, and SQ 13% in volume. ^c^ Calculated values based on the measured partition coefficients and composition ratios of the four sebum components.

**Table 3:** Summary of human volunteer experiments and values of *D*_SC_ estimated by the present model.

Reference	Experimental Condition						*D*_SC_ (m^2^ s^−1^)	
	Number of Subjects	Radon Activity Concentration (Bq m^−3^)		Water Temperature (°C)	Bath Time (min)	Breath Sampling for Radon Measurement		
		Water	Air					
Nagy [16] ^a^	17 (Male 8; Female 9)	Average: 73 × 10^3^ Range: (65–86) × 10^3^	<1.9 ^b^	31	60	Once immediately after bathing.	5th percentile Mode GM ^c^ (median) AM ^c^ 95th percentile	9.7 × 10^−15^ 1.6 × 10^−14^ 6.9 × 10^−14^ 1.4 × 10^−13^ 5.0 × 10^−13^
Tempfer et al. [17]	1 (Female)	950 × 10^3^	3000	37	20	Eleven times during and after bathing.	Best estimate	2.5 × 10^−14^
Furuno [18] ^a^	1 (Unknown)	58 × 10^3^	274	36	60	Five times during bathing.	Best estimate	1.5 × 10^−13^


^a^ Since it was impossible to identify which data corresponded to male or female subjects, the analyses were performed individually assuming that the subjects were all male or female. The sex averaged values of *D*_SC_ are represented here. ^b^ Radon-free air was used for breathing. ^c^ GM, geometric mean; AM, arithmetic mean.

## Data Availability

Not applicable.
